# Nosocomial Acquisition of Dengue

**DOI:** 10.3201/eid1010.031037

**Published:** 2004-10

**Authors:** Dirk Wagner, Katja de With, Daniela Huzly, Frank Hufert, Manfred Weidmann, Susanne Breisinger, Sabine Eppinger, Winfried Vinzent Kern, Tilman Martin Bauer

**Affiliations:** * University Hospital, Freiburg, Germany

**Keywords:** dengue virus, patient-to-professional disease transmission, reverse transcriptase polymerase chain reaction, enzyme-linked immunosorbent assay, needlestick injuries, dispatch

## Abstract

Recent transmission of dengue viruses has increased in tropical and subtropical areas and in industrialized countries because of international travel. We describe a case of nosocomial transmission of dengue virus in Germany by a needlestick injury. Diagnosis was made by TaqMan reverse transcription–polymerase chain reaction when serologic studies were negative.

Dengue viruses are transmitted by *Aedes* mosquitoes in tropical regions worldwide. The global incidence of epidemic and endemic dengue fever has increased substantially and is estimated at 50–100 million cases per year. International travel leads to imported cases in countries of the Northern Hemisphere (*1*), where dengue fever is an important differential diagnosis of fever in travelers returning from the tropics. Occupational needlestick injuries continue to pose a substantial risk for healthcare workers and occur at rates of 1.0 to 6.2 per 100 person-years (*2*). Common concerns are the transmission of HIV, hepatitis B virus, and hepatitis C virus. However, other pathogens can be transmitted as well. We report a case of nosocomial transmission of dengue virus.

## The Study

The index patient, a 26-year-old woman, was admitted to the infectious disease ward of a university hospital with a temperature of 40°C and myalgias 3 days after she returned from a 3-week trip to Cambodia and Thailand. Dengue virus infection was subsequently diagnosed, and mild hepatitis and a rash developed. She was discharged in good condition after the fever subsided. On the day of admission of the index patient (day 0_i_), a nurse sustained a needlestick injury with a hollow needle that had been used for drawing blood from the index patient. The needlestick resulted in a bleeding puncture wound that was immediately treated with an antiseptic. The index patient did not report any high-risk activity for HIV or hepatitis B virus, and the nurse had been immunized against hepatitis B virus. Therefore, no specific postexposure prophylaxis was performed. The nurse had previously been in good health and had not traveled outside Germany in the preceding 12 months.

Four days after the needlestick, headache, myalgias, and arthralgias developed in the healthcare worker, for which she took ibuprofen. Seven days later, when she was experiencing an intense headache and noticed a macular rash on her trunk, she sought treatment from a local doctor (day 0_n_). Physical examination showed bilateral cervical lymphadenopathy. On day 2_n_, she visited our service, where dengue virus infection was diagnosed by using a Light Cycler (Roche Diagnostics, Mannheim, Germany) polymerase chain reaction (PCR) method. Her symptoms lessened gradually over the course of 4 weeks, and she was on sick leave for 5 weeks. The time frame of the respective clinical presentation and the virologic results of the index patient and the nurse are shown in the Figure; laboratory data are presented in the Table.

**Figure:**
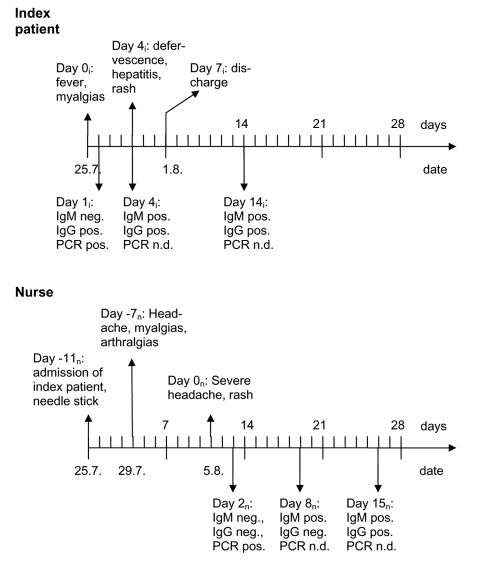
Time line of the signs, symptoms, and diagnostic tests in the index patient (i) and nurse (n). Ig, immunoglobulin; PCR, polymerase chain reaction; n.d., not done.

**Table:** Laboratory data for index patient and health care worker infected with dengue virus^a^

	Day	Leukocytes (mL)	Lymphocytes (mL)	Thrombocytes (mL)	Dengue IgM EIA	Dengue IgG EIA (U)	Dengue PCR
Index patient	0_i_	2.000	420	137.000	–	+ (16.4)	+
	4_i_	1.900	ND	55.000	+	+ (43.0)	–
	14_i_	5.000	1.650	375.000	+	+ (30.9)	ND
Health care worker	2_n_	2.600	590	136.000	–	– (2.8)	+
	8_n_	4.000	1.040	174.000	+	– (5.9)	ND
	15_n_	4.200	1.190	213.000	+	+ (16.4)	ND
	22_n_	4.800	1.220	215.000	+	+ (21.7)	ND

^a^Ig, immunoglobulin; EIA, enyzyme immunosorbent assay; PCR, polymerase chain reaction; ND, not done.

Serologic studies were performed with the PanBio dengue immunoglobulin (Ig) M capture enzyme-linked immunosorbent assay (ELISA) and PanBio dengue indirect IgG ELISA (PanBio Ltd., Brisbane, Australia) (*3*); arbitrary units relative to a simultaneously measured calibrator >11 were considered positive. For detecting virus RNA, RNA was prepared from 140 µL of serum by using the QIAamp Viral RNA Mini Kit (Qiagen, Hilden, Germany), according to the manufacturer’s instructions. To detect specific dengue virus RNA, we adapted a TaqMan-reverse transcription (RT)-PCR (*4*) to detect any of the four serotypes by using the following: degenerated forward primer (DEN FP), reverse primer (DEN RP); and probe (DEN P): DEN FP 5´AAggACTAgAggTTAKAggAgACCC3´, DEN RP 5´ggCCYTCTgTgCCTggAWTgATg3´ and the probe DEN P 5´ FAM-AACAgCATATTgACgCTgggARAgACC-TAMRA-3´. RT-PCR conditions for the Light Cycler (Roche Diagnostics) were: RT at 61°C for 20 min, activation at 95°C for 5 min, and 40 cycles of PCR at 95°C for 15 s, 60°C for 60 s. We used the RNA Master Hybridization Probes Kit (Roche Diagnostics) with 500-nM primers and 200-nM probes. The kit includes an aptamer-blocked *Thermus thermophilus* DNA polymerase, which performs RT and, once the aptamer drops out at activation, hotstarts PCR amplification.

## Conclusions

This is the fourth reported case, to our knowledge, of nosocomial dengue virus transmission (*5**–**7*) and the first in which TaqMan RT-PCR was used to provide evidence of nosocomial transmission before the detection of an antibody response. The index patient had acquired a dengue virus infection in Southeast Asia and experienced typical symptoms. In particular, she was febrile on admission, when the needlestick injury of the nurse occurred. In the health care worker who sustained the injury, cephalgia and myalgias developed after an incubation period of 4 days. A typical rash appeared after 11 days, when she also had a severe headache. The absence of fever, the most common sign of dengue fever, is likely due to the administration of ibuprofen. Both persons completely recovered. However, the healthcare worker was on sick leave for 5 weeks with resulting socioeconomic consequences.

The diagnosis was confirmed in both cases by both seroconversion and detection of dengue viral RNA by TaqMan RT-PCR; the latter gave positive results in both cases 3 and 6 days, respectively, before serum specimens were shown to contain antibody. Dengue viremia is known to correlate well with the presence of fever (*8*), which was the case in the index patient. Our report illustrates the potential of percutaneous nosocomial transmission of dengue viruses. This risk is likely to increase with the increase in the number of dengue infections imported to countries where dengue viruses are not endemic.
